# Angular dependence of the output of a kilovoltage X‐ray therapy unit

**DOI:** 10.1120/jacmp.v11i3.3216

**Published:** 2010-06-09

**Authors:** Mehran Goharian, Ian Nygren, Mauro Tambasco, Jose Eduardo Villarreal Barajas

**Affiliations:** ^1^ Medical Physics Department, Tom Baker Cancer Centre University of Calgary; ^2^ Department of Oncology, Faculty of Medicine University of Calgary; ^3^ Department of Physics and Astronomy University of Calgary Calgary Alberta Canada

**Keywords:** kilovoltage X‐ray therapy, quality control, orthovoltage, superficial

## Abstract

During the recommissioning of a Philips RT‐250 kilovoltage X‐ray unit, unexpected output variations with tube head rotation (cross‐plane) and tube head tilt (in‐plane) were observed. The measured output showed an increase of up to 7.3% relative to the neutral position (0° in‐plane and 0° cross‐plane) over the possible range of angles of in‐plane rotation for 75 kVp (half‐value layer, HVL=1.84 mm Al). A less pronounced but noticeable output change (with respect to the neutral position) was observed for cross‐plane rotation reaching 2% for the 225 kVp beam (HVL=0.90 mm Cu). This output variation was observed while manually adjusting the current to maintain constancy according to the current meter gauge. In order to address the observed output dependence with head orientation, the dose rate monitor chamber of the kilovoltage unit was calibrated to monitor the beam output in real time. The dose rate was manually adjusted to maintain a constant dose rate (in r/min) as displayed on the r/min gauge. This approach resulted in maintaining beam output for the 75 kVp and the 225 kVp beams within ±2% for the in‐plane angle variation and ±0.5% for the cross‐plane angle variation. A daily output check that includes ion chamber‐based measurements at the neutral position and an in‐plane angle of 45° has been implemented using the constant dose rate approach to monitor the stability of the X‐ray beams. As a result of the output variations with in/cross‐plane rotation, the quality control (QC) procedures that are typically used for clinical setup have been modified to test the stability of the beams under the non‐neutral positioning of the X‐ray tube.

PACS number: 87.56.Fc

## I. INTRODUCTION

Kilovoltage X‐ray therapy is commonly used for a wide range of superficial cancers. The typical cases treated on these units include basal, squamous and markel cell carcinomas. Both radiation prescription and equipment calibration can affect the treatment results. A quality control (QC) protocol is necessary to ensure proper machine performance during operation. The key control parameters in daily and monthly QC for orthovoltage and superficial X‐ray units include output constancy, beam quality, beam symmetry, timer accuracy and linearity, and identification and integrity of filters and filter interlocks.^(^ ^1^ ^)^ A regular daily output constancy check will help monitor the stability of machine output and therefore ensure that the intended prescribed dose is delivered accurately. Aside from dose accuracy, the daily output check allows physicists to monitor the machine output performance so that corrective actions can be taken to address any deviations outside/larger than the predefined action levels.^(^ ^1^ ^)^ At present, no specific recommendations for checking output constancy at different X‐ray tube head tilt (in‐plane) and tube head rotation (cross‐plane) positions are given by any of the most commonly used dosimetry protocols on standards for orthovoltage and superficial X‐rays.^(^ ^1^ ^–^ ^4^ ^)^ The purpose of this work is to report our finding regarding output dependency on in‐plane and cross‐plane rotation angles for the Philips RT‐250. It is worth mentioning that patients are typically treated in such a way that the X‐ray unit needs to be oriented to accommodate patient comfort, keep normal incidence, and eliminate unnecessary stand‐off between the patient and the cone. Therefore, the output dependency on tube head tilt and rotation is an important issue. To date, the authors are unaware of any published work discussing the output dependency on the tube head rotation of a kilovoltage radiotherapy unit.

## II. MATERIALS AND METHODS

**Figure 1:**
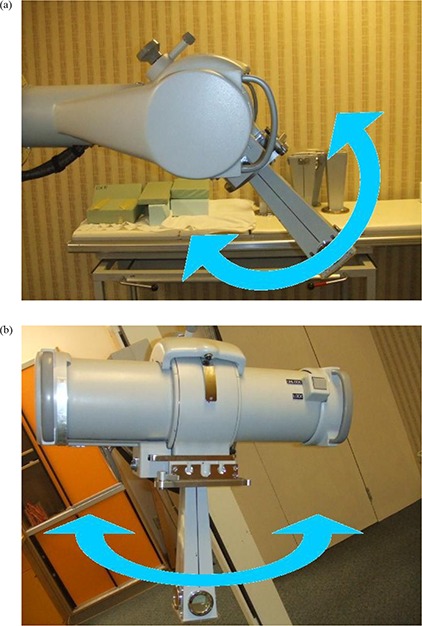
X‐ray tube head tilt (in‐plane) (a) and head rotation (cross‐plane) (b) orientations.

**Figure 2:**
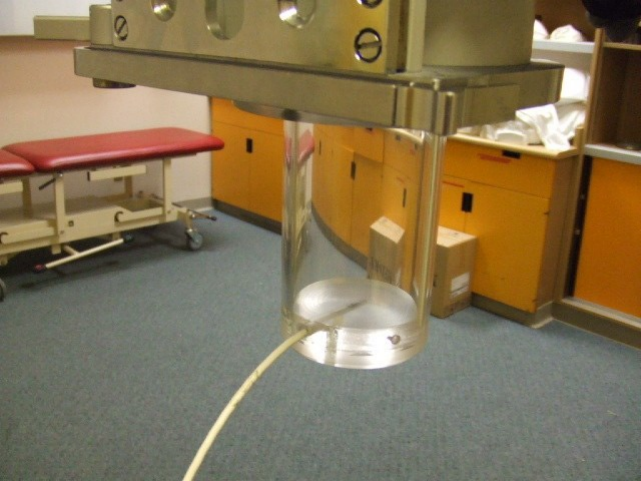
The ion chamber and holder were used for output consistency check

The kilovoltage unit, Philips RT‐250 (Philips Medical Systems, Andover, MA), has three degrees of motion: in‐plane (−30° to 90°), cross‐plane (−90° to 90°) (Fig. 1) and vertical. The machine has a sealed ionization chamber for monitoring beam dose rate. This chamber is set to give a 60 r/min reading when a test signal (current) is injected. This check is performed to verify the correct operation of the beam monitor chamber. Filters of various thicknesses and materials are available which, in combination with the tube high voltage, define the beam quality. The Philips RT‐250 uses fixed applicators with open (for 75 kVp beam) and closed (for 225 kVp beam) ends to define the prescribed field. Both the 75 kVp and 225 kVp beams, with added filtration of 2.0 mm Al and 0.35 mm Cu, respectively, were studied. Absolute dosimetry was performed according to the “in‐air” protocol of American Association of Physicist in Medicine (AAPM TG‐61) for both energies.^(^ ^3^ ^)^ A $0.6\;{\text{cm}}^{3}$ cylindrical ion chamber (Capintec model PR‐06G, Capintec, Ramsey, NJ) along with an electrometer (Capintec, model 192) was used for all absolute dose measurements. For the daily output constancy check, a $0.15\;{\text{cm}}^{3}$ cylindrical ion chamber (Capintec model PR‐05P) mounted in an acrylic jig (Fig. 2) was cross‐calibrated with the $0.6\;{\text{cm}}^{3}$ reference chamber. The X‐ray beam output was monitored at different in‐plane and cross‐plane tube head angles either by manually maintaining a constant current (mA) or a constant dose rate(r/min) as displayed on the gauges located on the front panel of the orthovoltage console. For these measurements, the reference chamber was positioned at the end of the reference cones (5 cm diameter for 75 kVp and $4\times 6\;{\text{cm}}^{2}$ for 225 kVp) keeping constant source to chamber (center) distances of 34 cm and 50 cm for the 75 kVp and 225 kVp beams, respectively. The dose rate was set at the neutral position (0° in‐plane and 0° cross‐plane) at the predefined dose rates of 40 r/min and 100 r/min for 75 kVp and 225 kVp beams, respectively. These dose rates of 40 r/min and 100 r/min are correlated to 20 mA and 17 mA as displayed on meter gauge, respectively. All output vs. tilt and rotation angle measurements were performed while maintaining either the current or the dose rate at their reference values (40 r/min or 20mA for 75kVp, 100 r/min or 17 mA for 225 kVp). The rationale for investigating the use of maintaining constant current or dose rate was to determine the most effective approach to achieve constant beam output. These dose rates of 40 r/min and 100 r/min gave an output that was normalized to 1.00 (corresponding to an absolute dose rate in water of 70.2 cGy/min and 88.8 cGy/min for 75 kVp and 225 kVp beams, respectively) and set as the reference.

## III. RESULTS

**Figure 3:**
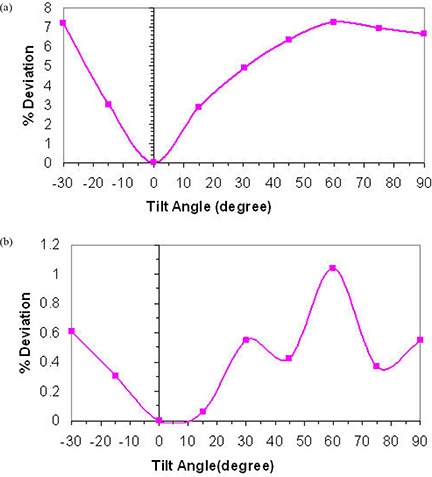
X‐ray beam output variation with head tilt angles for 75 kVp (a) and 225 kVp (b) using current meter to adjust output.

**Figure 4:**
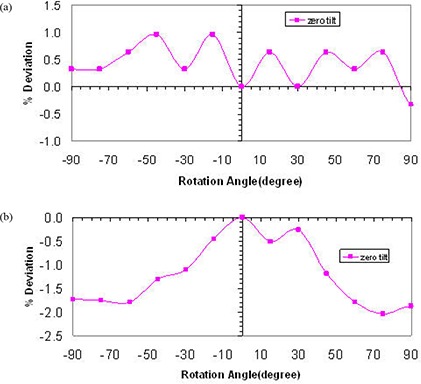
X‐ray beam output variation with head rotation angles at zero head tilt for 75 kVp (a) and 225 kVp (b) using current meter to adjust output.

**Figure 5:**
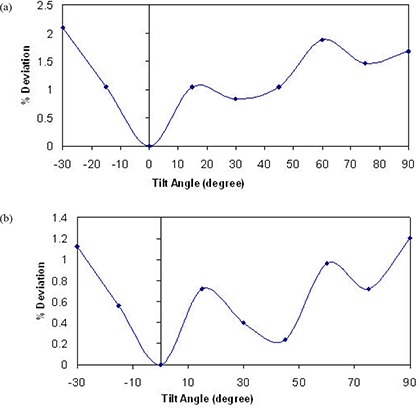
X‐ray beam output variation with head tilt angles for 75 kVp (a) and 225 kVp (b) using dose rate meter to adjust output.

**Figure 6:**
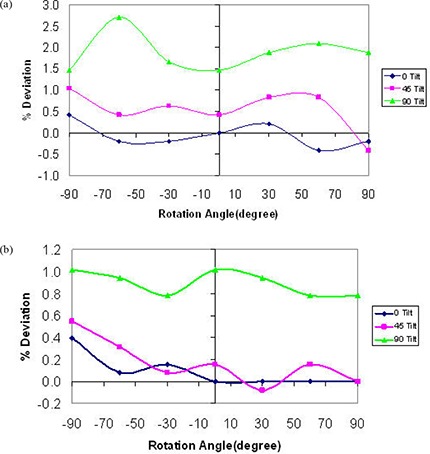
X‐ray beam output variation with head rotation angles for three different head tilt angles for 75 kVp (a) and 225 kVp (b) using dose rate meter to adjust output (dose rate maintained at 40 r/min and 100 r/min for 75 kVp and 225 kVp, respectively).

Figure 3 shows the percent deviation in beam output as a function of in‐plane angles for both 75 kVp and 225 kVp X‐ray beams while maintaining a constant current of 20 mA and 17 mA, respectively. These results indicate a dependence of the beam output on the tube head tilt that reaches 7.3% and 1.2% over the possible range of angles for 75 kVp and 225 kVp, respectively. It is clear that the deviation increases with increasing tube head tilt (away from the neutral 0° position) at both beam energies. Figure 4 indicates the percent deviation in beam output as a function of cross‐plane angles for both 75 and 225 kVp X‐ray beams, using the current meter to adjust the output. These results show a smaller output variation than observed for in‐plane rotations. However, the output can be lower than that observed at the neutral position (up to −2%) for the 225 kVp beam (Fig. 4(b)). It is worth noting that the variations in output with head tube rotation and tube head tilt were repeated over the course of four weeks, and each measurement point is the average of three readings. The maximum deviation of the readings respect to their mean value was within ±0.5%. Figures 5 and 6 show the percent deviation in beam output as a function of in/cross‐plane angles for both the 75 kVp and 225 kVp X‐ray beams while the output was kept constant by manually adjusting the dose rate to the predefined 40 r/min and 100 r/min. The output dependence with head tilt was reduced significantly from a maximum variation of 7.3% (using the constant current approach) to within 2.1% for the 75kVp beam. However, no significant differences on output variation were observed for the 225 kVp beam when the constant dose rate approach was used instead of the constant current (Figs. 3(b) and 5(b)).

For cross‐plane rotation, the beam output variation was checked for different in‐plane orientations, and it was found to vary up to 2.7% for the 75 kVp beam over the possible range of angles (Fig. 6(a)). For the 225 kVp beam, the output variation was constrained to 1% as shown in Fig. 6(b).

## IV. DISCUSSION

In response to the output variation with tube head and tilt, we investigated potential electrical and mechanical issues that may be causing the observed effect. A major maintenance was performed. This maintenance included the replacement of the high voltage contacts on the head that allow the X‐ray tube to maintain the beam during tube head rotation and tilt, and the refurbishment of the oil pump followed by a complete check of the beam generator. The half value layer, focal spot, head leakage and absolute dosimetry measurements were performed after the maintenance. No significant differences were found relative to the premaintenance measurements (beam quality and output were unchanged). After this electrical and mechanical maintenance, the dose rate chamber was used to manually maintain the output by keeping the dose rate chamber readings at 40 r/min and 100 r/min for the 75 kVp and the 225 kVp beams, respectively, regardless of the tube head tilt/rotation position. Once this manual adjustment using the dose rate meter was implemented, a significant improvement in terms of beam stability was observed (Fig. 5; maximum output variation was reduced from 7.3% to 2.1% for 75 kVp). It is worth mentioning that the observed variation in output with tube head tilt and rotation for the 75 kVp and the 225 kVp beams while the current kept constant was consistently the same (within ±0.5%) when repeated after the maintenance. Therefore, the major maintenance did not change the beam output stability behavior. Because of the consistency of the output variation with tube head rotation and tube head tilt before and after the major maintenance, and for consistency, it was decided to include only measurements performed after the maintenance (see Figs. 3 to 6).

To investigate the causes of the unexpected output variations that occur with tube head tilt while the dose rate is kept constant, a set of filtered and unfiltered ionization chamber‐based measurements was performed. These measurements were performed using a special acrylic cone set with a slot to accommodate a metal filter (1.8 mm Al and 0.9 mm Cu for 75 kVp and 225 kVp, respectively) 30 cm upstream from the chamber (Capintec model PR‐06G). The ratio of filtered to unfiltered chamber measurements using a 0.5 minute time interval were made for in‐plane angles from −30° to 90° in 15° increments. This ratio was used as a surrogate for the beam quality. A small reduction in the ratio of filtered to unfiltered with respect to the neutral position was observed for both beams (1.3% and 0.6% on average for 75 kVp and 225 kVp, respectively). This reduction on the beam quality may amplify the chamber response, explaining the observed increase in output with head tube tilt.

## V. CONCLUSIONS

Kilovoltage radiotherapy machines should be calibrated following standard protocols, and the performance of machines needs to be constantly monitored under a QC program.^(^ ^5^ ^)^ In addition, an external dosimetric quality audit for kilovoltage radiotherapy equipment is recommended. This is particularly important since treatments with kilovoltage X‐ray units are typically hypofractionated. The observed output dependence with tube head orientation was decreased by using the dose rate monitor chamber instead of current meter to monitor the beam output. QC for a kilovoltage X‐ray therapy unit should include output stability with tube head tilt/rotation. Due to the possibility of output dependency with in‐plane rotation, the authors recommend that the daily output is monitored in at least two in‐plane rotation angles (e.g. 0° and 45°) using an ion chamber and holder (e.g. Fig. 2) as part of a routine daily QC procedure. Monitoring daily beam output using an independent ion chamber and electrometer system also provides a check on the performance of the kilovoltage therapy unit's monitor chamber. Monthly QC procedures have been adapted to monitor output at four in‐plane tube head rotation angles. For annual QC, a complete output variation with in‐plane and cross‐plane rotation angles is recommended.
